# Supplementary material for: Biomimetic Design for a Dual Concentric Porous Titanium Scaffold with Appropriate Compressive Strength and Cells Affinity
Source: Materials (Basel). 2020 Jul 25;13(15):3316. doi: 10.3390/ma13153316 (PMC7435892; doi:10.3390/ma13153316)
Supplement: Supplementary file 1 [file materials-13-03316-s001.pdf]

materials

Supplementary Materials: Biomimetic Design for a Dual Concentric Porous Titanium Scaffold with Appropriate Compressive Strength and Cells Affinity

Han Lee [1], Jiunn-Der Liao [1,2,*], Yao-Sheng Guo [1] and Yung-Der Juang [3]

[1] Department of Materials Science and Engineering, National Cheng Kung University, 1 University Road, Tainan 701, Taiwan; rick594007@hotmail.com (H.L.); sunrise10727@gmail.com (Y.-S.G.)
[2] International Center for Wound Repair and Regeneration, National Cheng Kung University, 1 University Road, Tainan 701, Taiwan
[3] Department of Materials Science, National University of Tainan, Tainan 700, Taiwan; juang@mail.nutn.edu.tw
* Correspondence: jdliao@mail.ncku.edu.tw; Tel.: +886-6-275-7575 (ext. 62971); Fax: +886-6-234-6290

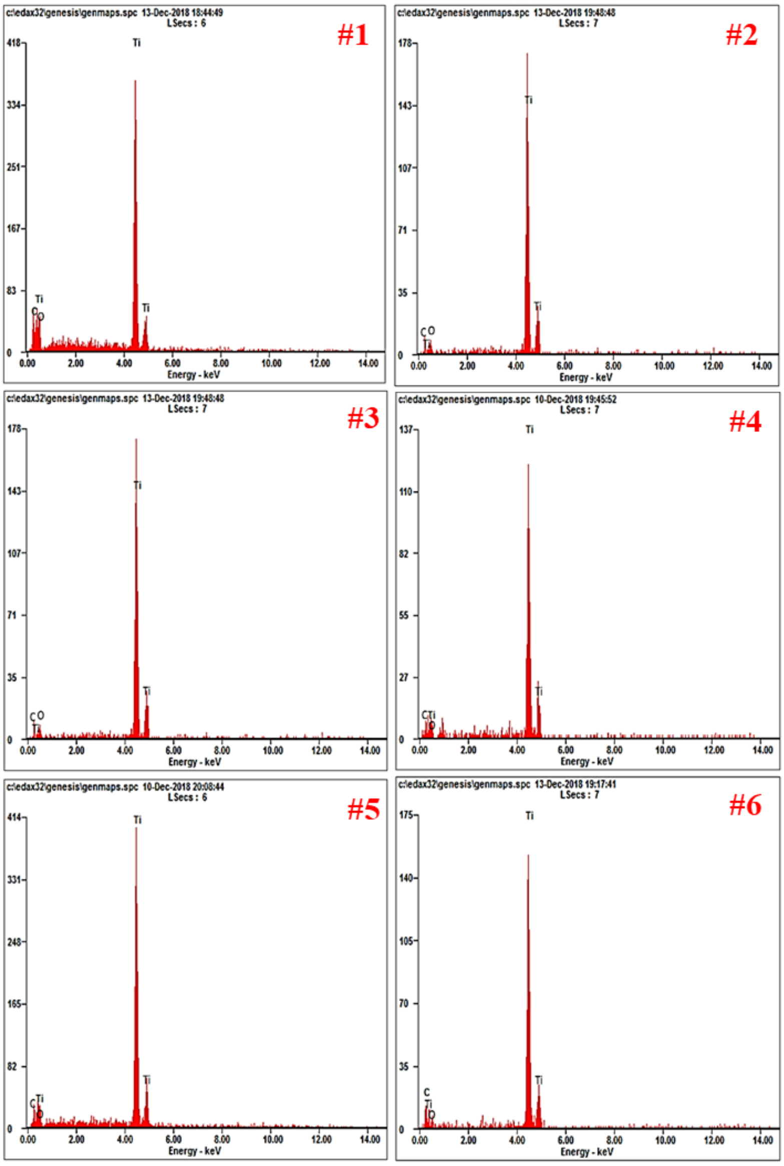

Figure S1. EDS data presenting the elements distribution upon the surfaces of (#1) CP-Ti, (#2) CP-Ti$_{10_45}$, (#3) CP-Ti$_{20_45}$, (#4) CP-Ti$_{20_55}$ (#5) CP-Ti$_{20_65}$, and (#6) CP-Ti$_{30_65}$.

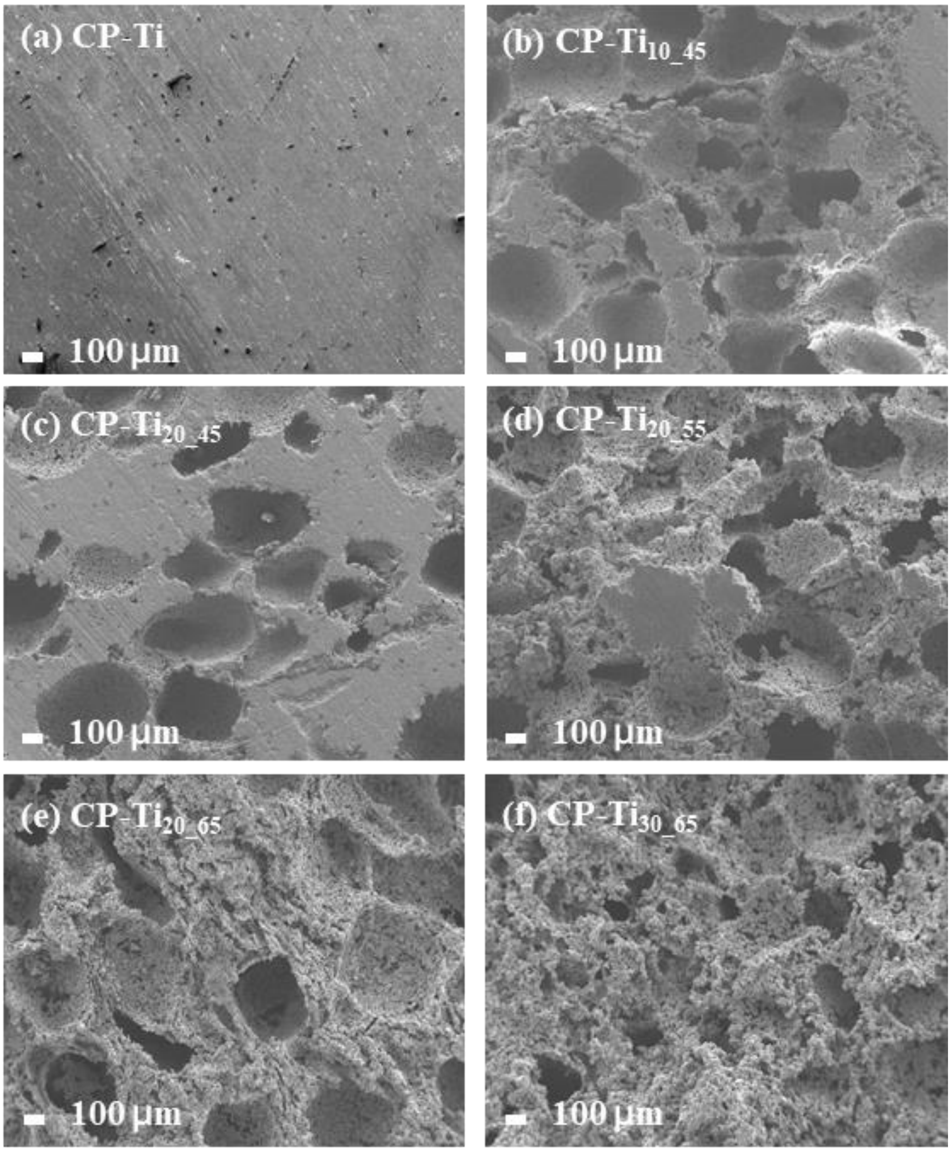

Figure S2. SEM photo-images showing pore sizes of dual porous structures on the surfaces of: (**a**) CP-Ti, (**b**) CP-Ti_{10_45}, (**c**) CP-Ti_{20_45}, (**d**) CP-Ti_{20_55} (**e**) CP-Ti_{20_65}, and (**f**) CP-Ti_{30_65}.

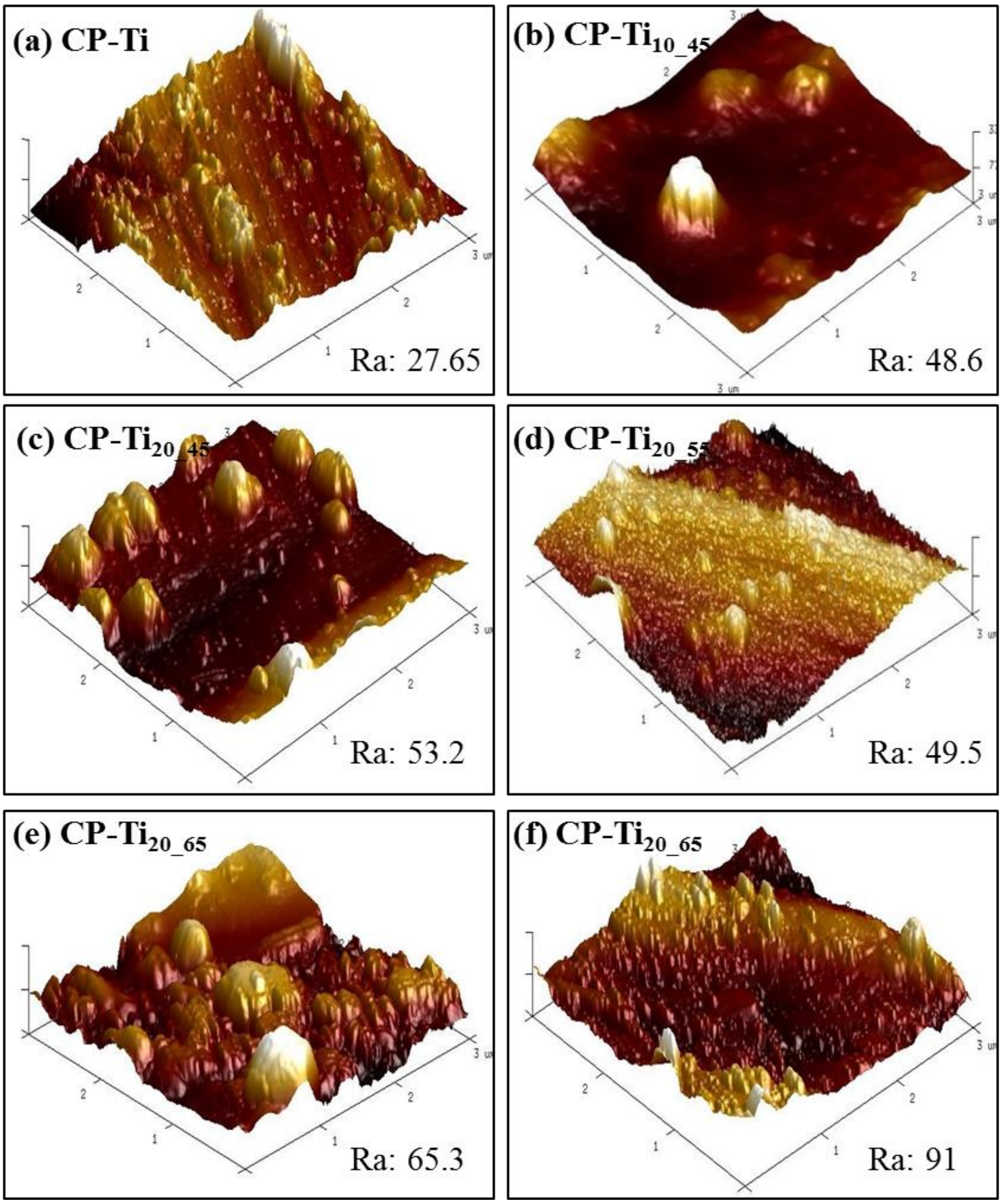

Figure S3. AFM topographical images illustrating for the surfaces of (**a**) CP-Ti, (**b**) CP-Ti$_{10_45}$, (**c**) CP-Ti$_{20_45}$, (**d**) CP-Ti$_{20_55}$ (**e**) CP-Ti$_{20_65}$, and (**f**) CP-Ti$_{30_65}$.

© 2020 by the authors. Submitted for possible open access publication under the terms and conditions of the Creative Commons Attribution (CC BY) license (http://creativecommons.org/licenses/by/4.0/).
